# Therapeutic ultrasound versus injection of local anesthetic in the treatment of women with chronic pelvic pain secondary to abdominal myofascial syndrome: a randomized clinical trial

**DOI:** 10.1186/s12905-022-01910-y

**Published:** 2022-08-02

**Authors:** Maria Carolina Dalla Vecchia Baltazar, Jéssica Aparecida de Oliveira Russo, Victória De Lucca, Andréia Moreira de Souza Mitidieri, Ana Paula Moreira da Silva, Maria Beatriz Ferreira Gurian, Omero Benedicto Poli-Neto, Júlio César Rosa-e-Silva

**Affiliations:** Department of Gynecology and Obstetrics, Ribeirao Preto Medical School, University of Sao Paulo, Av. Bandeirantes 3900, 8°Andar, Ribeirão Preto, 14049-900 Brazil

**Keywords:** Pelvic pain, Myofascial pain syndrome, Ultrasonic therapy, Local anesthesia

## Abstract

**Background:**

Chronic pelvic pain (CPP) is defined as recurrent or continuous pain in the lower abdomen or pelvis, either non-menstrual or noncyclical, lasting for at least 6 months. There is strong evidence that up to 85% of patients with CPP have serious dysfunctions of the musculoskeletal system, including abdominal myofascial pain syndrome (AMPS). AMPS is characterized by intense and deep abdominal pain, originating from hyperirritable trigger points, usually located within a musculoskeletal band or its lining fascia. In the literature, there are few studies that address AMPS.

**Objectives:**

To evaluate and compare the efficacy of therapeutic ultrasound (TUS) and injection of local anesthetic (IA) to improve pain in women with abdominal myofascial syndrome secondary to CPP.

**Study design:**

Randomized controlled clinical trial.

**Setting:**

Tertiary University Hospital.

**Materials and methods:**

A randomized clinical trial was conducted, patients were allocated to two types of treatment: group TUS (n = 18), and group IA (n = 20). The instruments used for evaluation and reassessment were the Visual Analog Scale, Numerical Categorical Scale, McGill Pain Questionnaire, and SF-36 quality of life assessment questionnaire. They were evaluated before starting treatment, 1 week after the end of treatment, and at 1, 3, and 6 months.

**Results:**

TUS and IA were effective in reducing clinical pain and improving quality of life through the variables analyzed among study participants. There was no significant difference between groups.

Limitations: absence of blinding; exclusion of women with comorbidities and other causes of CPP, the absence of a placebo group, the difference between the number of sessions used for each technique, and the COVID-19.

**Conclusion:**

Treatment with TUS and IA were effective in reducing clinical pain and improving quality of life in women with AMPS secondary to CPP.

**Trail registration:**

We declare that this clinical trial has been registered under the number [(ReBEC) no. RBR-39czsv] on 07/18/2018 in the Brazilian Registry of Clinical Trials.

## Background

Chronic Pelvic Pain (CPP) is defined as perceived painful symptoms originating from organs/structures pelvic pain, typically lasting more than 6 months. It is often associated with negative consequences from a cognitive, behavioral, emotional and sexual point of view, as well as with symptoms suggestive of urinary, intestinal, pelvic floor dysfunction, myofascial or gynecological [1]. Chronic pelvic pain is considered an important problem that affects women's quality of life [2]⁠.

Studies have frequently demonstrated the importance of the musculoskeletal system in the genesis and perpetuation of CPP [3–6]. In a cross-sectional study, abnormal pelvic musculoskeletal injuries were significantly more common among women with CPP compared with healthy women (Tu et al. 2008). There is evidence that up to 85% of patients with CPP have serious dysfunction of the musculoskeletal system, thus, musculoskeletal conditions among women with CPP are common and need to be further recognized⁠. ⁠. Among them, abdominal myofascial pain syndrome (AMPS) appears to be one of the main symptoms [7–9]. It was observed in our previous study that approximately 30% of women seen at a pelvic pain outpatient clinic at a tertiary university hospital diagnosed with CPP have abdominal myofascial syndrome, and this has a direct relationship with those who underwent surgical procedures (particularly cesarean section) [5, 10]⁠.

AMPS is characterized as intense and deep abdominal pain, originating from hyperirritable trigger points (MTrPs) in the abdominal muscles, which can be classified as either active or latent [11–13]. Active MTrPs can cause pain at rest and produce referred pain similar to that felt during stitch compression, regardless of whether they are stimulated. Conversely, stimulation of these active points can promote activation of other latent trigger points that are sometimes located in regions distant from the active trigger point being stimulated. Latent MTrP, on the other hand, does not cause spontaneous pain, but can restrict movement or cause muscle weakness and only become painful if direct pressure is applied on them [14]⁠. The diagnosis is made through a combination of criteria: hypertonic point in a set of muscle fibers, recognition of pain on palpation of the stitch, referred pain pattern, muscle contracture as a local response to palpation of the stitch (positive Carnett test), and limited range of motion [15–17]. The precise mechanism of the appearance and perpetuation of MTrP is still unknown, but it is believed that facilitating the release of acetylcholine in the plates terminal motor, with the release of neuronal substances, results in depolarization and, consequently, in the sustained contraction of muscle fibers [18]⁠. This sustained contraction results in a localized ischemia that, in turn, causes the release of histamine, serotonin, neurokinins, and prostaglandins that stimulate nociceptors, increasing the release of acetylcholine and generating reflex muscle contraction, resulting in a sustained cycle of pain and muscle spasm [19, 20]. Another explanation would be that the transient overload of a muscle could cause injury to the sarcoplasmic reticulum, and once the T tubule system is impaired, the stored calcium ions are released and recaptured in the area of the injury causing permanent fiber contraction [21]⁠. In addition to pain phenomena, MTrP can also produce muscle spasms and autonomic phenomena such as piloerection, vasoconstriction, hyperhidrosis, temperature changes, and a variety of somatovisceral reflexes [12, 14].

The treatment of AMPS requires a multidisciplinary approach to interrupt the pain cycle, abolish MTrP, and restore muscle flexibility, eliminating the factors of predisposition and perpetuation of pain [18]⁠. In addition to systemic pharmacological treatments, including analgesics, muscle relaxants, antidepressants, and nonsteroidal anti-inflammatory drugs, specific therapies such as ischemic compression have been proposed [14, 22]. The most used forms of electrotherapy in AMPS treatment are transcutaneous electrical nerve stimulation (TENS), interferential current stimulation ⁠[14], and acupuncture [23–25]⁠. Anesthetic injection, in the active trigger points, is the most used treatment and has been recommended as an effective technique for the treatment of symptoms related to the presence of active trigger points. Its application can be performed under short action, prolonged action, or through a combination of local anesthetics, such as 1% lidocaine and 0.25% bupivacaine [26, 27]. Some authors indicate the administration of 2–4 mL of 1–2% lidocaine directly at the trigger point ⁠[14, 28, 29]. Therapeutic ultrasound (TUS) is a modality of longitudinal sound energy of deep penetration, which, when transmitted to biological tissues, can produce cellular changes by mechanical effects [30]⁠⁠. In ultrasound therapy, the intensity used is between 0.1 and 3 w/cm^2^, but for the treatment of a trigger point in myofascial pain syndrome, the most often used intensity is from 1 to 1.5 w/cm^2^. The application time for MTrP with TUS in myofascial syndrome varies from 4 to 10 min [31–33]⁠.

Although local anesthetic injection is considered the gold standard in the treatment of myofascial syndromes, it is noted that some women do not respond to it [5]. In the literature, there are few studies that address abdominal myofascial syndrome [3, 5, 7–10, 34–36] and there are no studies on the use of therapeutic ultrasound for the treatment of this syndrome. However, its use is known to be effective in trapezius muscle trigger points [31]. Considering this gap, our objective was to evaluate and compare the effectiveness of therapeutic ultrasound and local anesthetic injection in improving pain and quality of life in women with abdominal myofascial pain syndrome secondary to chronic pelvic pain.

## Material and methods

### Study design

An experimental study was conducted through a randomized clinical trial, on 38 women, with 18 participants in the TUS group and 20 participants in the local anesthetic injection group. The study was approved by the Research Ethics Committee of the University of Sao Paulo, and informed consent was obtained (no. CAAE 80822717.1.0000.5440). It was prospectively registered in the Brazilian Registry of Clinical Trials [(ReBEC) no. RBR-39czsv] on 07/18/2018.

### Participants

The first study participant was recruited on 07/22/2018.

#### Eligibility criteria

We included women between 18 years of age and non-menopausal women with a clinical diagnosis of abdominal myofascial syndrome with the presence of only one active trigger point and pain above 4.4 (moderate pain) on the Visual Analog Scale (VAS) and without previous treatments.

Women who present with the following were excluded: pregnant women, with hip prostheses, neoplasms in the abdominal-pelvic region, severe osteoporosis, copper intrauterine device, abdominal varicose veins, cognitive deficits that make it difficult to understand the questionnaires, women with anticoagulation or hemorrhagic disorders, and local or systemic infections, allergies to anesthetics, acute muscle trauma, extreme fear of needles, and history of complaints of chronic musculoskeletal pain, such as fibromyalgia, chronic fatigue, or diabetes. Exclusion criteria also included those who used chronic pain relievers, anti-inflammatory drugs, tricyclic antidepressants, and aspirin within 3 days before the injection. All patients with suspected interstitial cystitis, irritable bowel syndrome, or other disease that justifies or contributes to CPP will also be part of the exclusion criteria for endometrioma or hernia as evidenced by ultrasound of the abdominal wall and abdominal wall infections, as well as women who went missing after starting treatment.

#### Settings and locations for data collection

The patients were recruited and treated at the CPP outpatient clinic of a tertiary university hospital. After AMPS confirmation, following the diagnostic criteria⁠⁠ [15, 16], patients who met the inclusion criteria were invited to participate in the study and were randomized. Patients then underwent assessments of abdominal trigger point, pain, and quality of life.

The active trigger point was measured using a tape measure, and to determine its exact location, the distance from the point to the various abdominal anatomical structures was measured. The clinical pain threshold was then assessed using the VAS, Numerical Categorical Scale (NCS) and McGill Pain Questionnaire. Quality of life was assessed using the SF-36 quality of life questionnaire.

After a sequence of evaluations, the patients started the TUS treatments or injection of local anesthetic, according to the results of the randomization process.

After the completion of treatments, participants were reevaluated with the same parameters at 1 week and 1, 3, and 6 months after intervention, and patients in both groups were instructed not to use central analgesics or nonsteroidal anti-inflammatory drugs for 72 h before the reevaluation.

##### Instruments used to assess patients

*Measuring pain* The instruments used in both stages have already been validated and are applicable in both scientific research and clinical applications. The clinical measurement of pain will be performed using one-dimensional and multidimensional scales [37]⁠⁠. The VAS of pain is the one-dimensional scale most commonly used in clinical practice because of its feasibility, speed, and clinical application, despite some criticisms of its linearity [38]⁠⁠. It consists of an uninterrupted line of 100 mm in length, in which the patient is instructed to mark the point that corresponds to the referred pain, remembering that the beginning of the scale (0) corresponds to the absence of pain and the end of the scale [10] corresponds to the worst pain already experienced (delivery without analgesia, myocardial infarction, toothache, urinary lithiasis, etc.) or imagined. It has the advantage of simplicity. The NCS for pain will also be used, in which the participant grades the pain in intervals from 0 to 5 (0, no pain; 1, mild; 2, uncomfortable; 3, distressing; 4, horrible; or 5, martyrizing) [39].

As for the multidimensional scale, the most important and widespread is the McGill Pain Questionnaire [40, 41]⁠⁠. It is widely accepted as reliable, valid, sensitive, and precise. It consists of a questionnaire of pain descriptors, grouped into four classes – sensory, affective, evaluative, and miscellaneous—and 20 subclasses. Despite the apparent complexity, it allows the patient to portray her painful experiences in more detail.

*Clinical measurement of quality of life* This measure is being carried out through the generic questionnaire to assess the quality of life Medical Outcomes Study SF-36, developed by the World Health Organization, translated and validated in Portuguese [42, 43]⁠ ⁠and widely used in the evaluation of chronic pain [44]⁠⁠. It consists of 36 items assessing functional capacity, physical aspects, pain, state of health, vitality, social aspects, emotional aspects, and mental health.

*Demographic data *Women were assessed for demographic data (age, parity, marital status, monthly family income, education level).

## Interventions

### Therapeutic ultrasound

At each session, patients underwent palpation examination of the active trigger point, previously measured for the realization of treatment with TUS at the same point at each session. Treatment with TUS was performed by a previously trained physical therapist, once a week, for 10 consecutive weeks, with a frequency of 1 MHz and intensity of 1 W/cm^2^ for 5 min in the region of the active trigger point [31, 33]⁠ To perform this treatment, a TUS device (Sonopulse III, Ibramed, Amparo, São Paulo), with a frequency of 1 and 3 MHz, was used. The therapeutic ultrasound equipment was calibrated once a year, as recommended by the manufacturer.

### Injection of local anesthetic

At each session, patients underwent palpation examination of the active trigger point, previously measured for the administration of lidocaine at the same point at each session. This procedure was performed by doctors with experience at the CPP outpatient clinic of the Hospital das Clínicas of the Ribeirão Preto Medical School of the University of São Paulo, and then 2 mL of 1% lidocaine was administered, without a vasoconstrictor [14, 28, 29]⁠, ⁠with a 22-gauge needle, measuring 0.70 mm × 0.25 mm (Injex Indústrias Cirúrgicas Ltda, Ourinhos-SP, Brazil), directly and perpendicular to the active trigger point. At the end of the application, direct compression with sterile cotton was applied for at least 2 min, to avoid the formation of a local hematoma [12, 14]⁠⁠. The treatment was performed once a week for 4 consecutive weeks, as standardized by the outpatient clinic [29].

## Sample calculation

Based on the objectives of this phase, the sample size calculation was performed to test two experimental proportions with samples of the same size (group of cases treated with lidocaine injection × group of cases treated with TUS, using the following expression:

$${\text{n}} = \frac{{_{{[{\text{z}}\alpha }} .\left( {{\text{p1}}.{\text{q1 }} + {\text{ p2}}.{\text{q2}}} \right)^{{{1}/{2}}} + {\text{ z}}_{{{1} - \beta }} .\left( {{\text{p1}}.{\text{q1 }} + {\text{ p2}}.{\text{q2}}} \right)^{{{1}/{2}}} ]^{{2}} }}{{\left( {{\text{p}}_{{2}} - {\text{ p}}_{{1}} } \right)^{{2}} }}$$

Based on the literature [26]⁠⁠, it was considered.

- p2 = 60%, considering an unsatisfactory result that is 30% lower.
- zα = 1.645, considering α = 5% and unilateral test.
- z1-β = 1.2815, considering test power (1-β) = 90%

Thus, we have a determined sample size that is equal to 22 individuals for each group, pointing out that the response rate is different between groups, with the conditions of significance and power of the test considered, at 5 and 90%, respectively. Based on this calculation, the proposal is to include 30 subjects in each group and make the first analyses of the test’s power and effectiveness when 50% of the group is included, that is, 15 individuals in each group. Women who agree to participate and meet the inclusion criteria will be invited to return to the clinic, and the choice of treatment will be established by drawing through a computer and placed in envelopes that will be opened at the time of the first appointment.

## Randomization

Randomization was performed through a sequence generated using an online tool for the two treatment groups, using blocks of random size of four (https://www.sealedenvelope.com/randomisation/internet/): group A, TUS, and group B, injection of local anesthetic. The JCRS researcher generated the random allocation sequence, and the MCDVB researcher enrolled the participants and designated the participants for the interventions.

## Blinding

There was no blinding in this study; however, the data analysis was performed by a third researcher blinded to the types of treatment.

## Statistical analysis

Analysis was performed by intention to treat. An exploratory data analysis was performed using measures of the central position and dispersion. Qualitative variables were summarized considering absolute and relative frequencies. Comparison between the groups regarding the quantitative variables was carried out using the Wilcoxon nonparametric test for independent samples. The chi-squared test was used to compare groups in the qualitative variables. Comparison between the times within each group and between the groups within each time point was performed considering the orthogonal contrasts in the mixed-effects regression model. The analyses were implemented in Statistical Analysis System (SAS) version 9.4 program.

## Ethics approval and consent to participate

The study was approved by the Research Ethics Committee of the University of Sao Paulo, and informed consent was obtained from all study participants (no. CAAE 80822717.1.0000.5440). Consent forms were obtained from all study participants, and that the patients were all over 18 years of age, and that they were literate and therefore no consent form was required to be signed by parents, guardians or legal representatives. We follow all the guidelines of the Declaration of Helsinki.

## Results

**Fig. 1:**
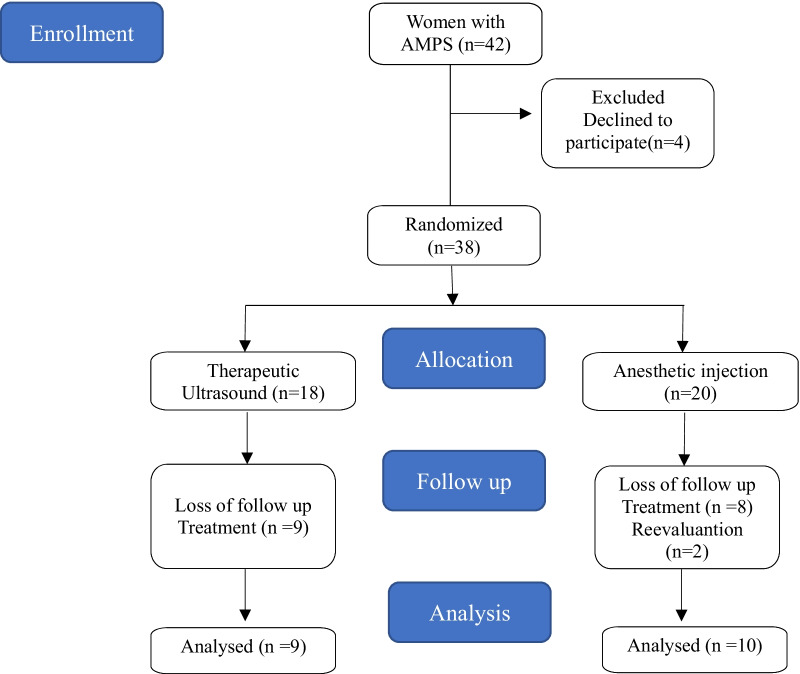
Flowchart of patient recruitment

Overall, 42 patients were screened for the study, four of these patients did not accept to participate because they were not available to attend the scheduled procedures. Thirty eight women were randomized for the study (TUS = 18; AI = 20): 19 patients completed the treatment and reevaluation protocol (TUS = 9; AI = 10); 2 patients completed the treatment but did not attend the reevaluations (TUS = 0; AI = 2), one at the 3 months post treatment and the other six months post treatment; 17 patients abandoned treatment (TUS = 9; AI = 8): 2 patients did not complete the treatment protocol due to the COVID-19 pandemic (TUS = 2; AI = 0), 5 patients dropped out due to treatment results – ​​worsening (TUS = 1; AI = 2) or improvement (TUS = 2; AI = 0) of symptoms with the onset of treatment and 10 patients abandoned treatment for reasons unrelated to the treatment itself, such as work, studies, family problems, and other health problems (TUS = 4; AI = 6) (Fig. 1). Patients were recruited from 06/15/2018 to 8/31/2020.

Table 1 shows all the variables analyzed, with no differences between groups. Thus, the sample was considered homogeneous (*P* > 0.05).

**Table 1:** Characterization of samples from groups IA, injection of local anesthetic, and TUS, therapeutic ultrasound

Variables	Group IA (n = 20)	Group TUS (n = 18)	P*
	Mean and SD		
Age (years)	42.20 (± 9.37)	39.89 (± 6.36)	0.9622
Weight (kg)	72.95 (± 12.97)	74.50 (± 16.32)	0.2057
Height (m)	1.60 (± 0.08)	1.61 (± 0.07)	0.2780
BMI	28.50 (± 4.91)	28.93 (± 6.67)	0.1232
Pain time (months)	90.85 (± 99.69)	81.78 (± 86.42)	0.0484
*Parity*			
No. of pregnancies	3.20 (± 2.12)	2.67 (± 1.75)	0.2412
Cesarean	1.45 (± 1.39)	1.11 (± 1.13)	0.4689
Vaginal birth	1.30 (± 1.84)	1.22 (± 1.77)	0.0247
Abortion	0.55 (± 0.89)	0.33 (± 0.49)	0.1765
*N–%*			
Marital status			0.6706
Married	12–60%	12–66.67%	
Single/divorced/widowed	8–40%	6–33.33%	
Education			0.1798
Up to 1st grade	11–55%	6–33.33%	
Up to 2nd grade	9–45%	12–66.67%	
Profession			0.7320
Unpaid	10–50%	10–55.56%	
Paid	10–50%	8–44.44%	
Location of MTrP			0.4626
Right	9–45%	6–33.33%	
Left	11–55%	12–66.67%	

Wilcoxon nonparametric test for independent samples

Chi-square test

IA, injection of local anesthetic; TUS, therapeutic ultrasound; SD, standard deviation; P *, p value; BMI, body mass index; N, sample number.

**Fig. 2:**
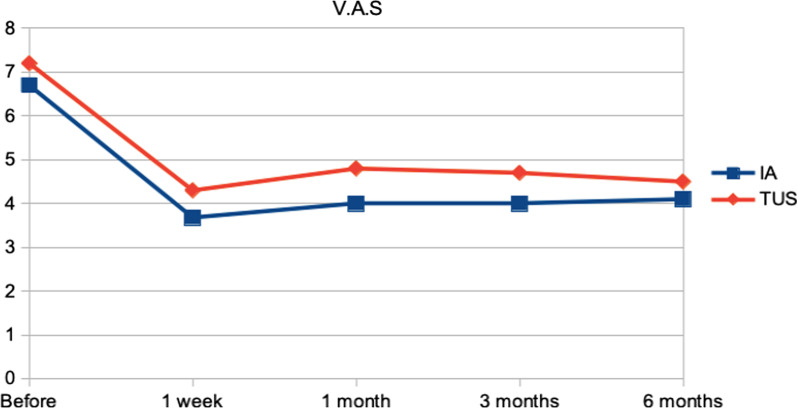
Comparison of clinical pain evolution using the Visual Analogue Scale between intervention groups

**Fig. 3:**
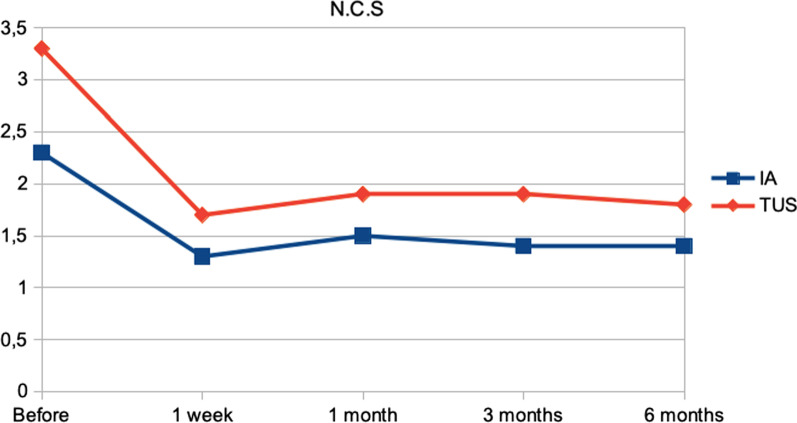
Comparison of the evolution of clinical pain using the Numerical Categorical Scale between the intervention groups

Tables 2 and 3 and Figs. 2 and 3 show the evolution of the response to the intervention of TUS and injection of local anesthetic. Clinical pain parameters were assessed using the McGill Pain Questionnaire (Table 2), Table 3, Figs. 2 and 3 show the evolution of clinical pain parameters assessed by VAS and NCS. The quality of life was assessed using the SF-36 questionnaire (Table 4).

**Table 2:** Evolution of clinical pain measured using the McGill Pain Questionnaire in the intervention groups

McGill	Total number of descriptors			Total pain index number		
	IA mean (SD)	TUS mean (SD)	P*	IA mean (SD)	TUS mean (SD)	P*
Before	13.40 (± 5.13)	15.33 (± 4.77)	0.4197	30.65 (± 12.30)	37.94 (± 13.74)	0.2325
1 week	9.50 (± 7.23)	9.11 (± 8.30)	0.8709	21.65 (± 15.87)	23.00 (± 22.62)	0.8247
1 month	8.10 (± 6.98)	10.00 (± 8.32)	0.4278	19.55 (± 16.96)	25.78 (± 22.49)	0.3077
3 months	8.25 (± 7.51)	10.44 (± 8.40)	0.3599	19.70 (± 17.94)	26.28 (± 22.30)	0.2814
6 months	9.05 (± 7.65)	9.33 (± 8.40)	0.9058	21.60 (± 17.92)	24.33 (± 23.04)	0.6539

Orthogonal contrasts in the mixed-effects regression model

*IA* injection of local anesthetic, *TUS* therapeutic ultrasound, *SD* standard deviation; P*, *p* value

**Table 3:** Mean and standard deviation of visual analog and numerical categorical scales to quantify clinical pain at assessment and reassessment times

Clinical pain	V.A.S			N.C.S		
	IA mean ± SD	TUS mean ± SD	P*	IA mean ± SD	TUS mean ± SD	P*
Before	6.70 (± 1.53)	7.22 (± 1.31)	0.6122	2.30 (± 0.86)	3.39(± 1.09)	0.0179
1 week	3.68 (± 2.77)	4.33 (± 3.85)	0.5470	1.35(± 0.93)	1.78 (± 1.80)	0.3483
1 month	4.00 (± 3.15)	4.89 (± 3.72)	0.3886	1.55 (± 1.32)	1.94 (± 1.73)	0.3871
3 months	4.00 (± 3.49)	4.78 (± 3.70)	0.4504	1.45 (± 1.32)	1.94 (± 1.73)	0.2786
6 months	4.10 (± 3.14)	4.50 (± 3.88)	0.6977	1.40 (± 1.10)	1.89 (± 1.81)	0.2840

Orthogonal contrasts in the mixed effects regression model

*IA* injection of local anesthetic, TUS therapeutic ultrasound, *V.A.S* visual analogue scale; *N.C.S* numerical categorical scale, *SD* standard deviation; P* *p*-value

**Table 4:** Evolution of quality of life measured using the SF-36 questionnaire in the intervention groups

SF-36						
	IA mean ± SD	TUS mean ± SD	P*	IA mean ± SD	TUS mean ± SD	P*
Domain	Functional capacity			Limitation by physical aspects		
Before	44.50 (± 22.71)	51.39 (± 26.56)	0.4167	17.50 (± 24.47)	19.44 (± 31.57)	0.8669
1 week	50.50 (± 24.54)	61.67 (± 26.90)	0.1888	23.75 (± 31.91)	33.33 (± 40.22)	0.4092
1 month	51.00 (± 24.42)	58.33 (± 25.50)	0.3873	21.25 (± 37.41)	30.56 (± 34.89)	0.4228
3 months	48.75 (± 28.14)	56.39 (± 25.08)	0.3679	26.25 (± 40.13)	25.00 (± 33.21)	0.9142
6 months	55.25 (± 28.77)	58.06 (± 27.23)	0.7406	40.00 (± 44.72)	25.00 (± 33.21)	0.1972
Domain	Pain			General health status		
Before	31.60 (± 15.26)	21.61 (± 16.61)	0.1352	45.35 (± 25.88)	47.44 (± 21.05)	0.7959
1 week	35.45 (± 25.95)	31.12 (± 22.75)	0.5205	47.65 (± 27.06)	53.28 (± 21.10)	0.4873
1 month	34.05 (± 20.27)	28.78 (21.37)	0.4291	44.30 (± 27.99)	51.00 (± 21.40)	0.4084
3 months	34.20 (± 19.91)	26.67 (± 20.51)	0.2591	48.30 (± 27.89)	48.44 (± 27.13)	0.9858
6 months	37.30 (± 19.81)	33.61 (± 25.93)	0.5799	48.75 (± 26.80)	51.72 (± 20.31)	0.7135
Domain	Vitality			Social aspects		
Before	32.00 (± 24.94)	31.67(± 21.42)	0.9684	41.65 (± 25.11)	40.06 (± 27.97)	0.8711
1 week	35.75 (± 26.22)	40.83 (± 26.25)	0.5456	45.95 (± 25.98)	49.78 (± 33.26)	0.6969
1 month	39.00 (± 23.54)	39.44 (± 25.95)	0.9578	51.05 (± 30.40)	47.72 (± 34.72)	0.7349
3 months	39.00 (± 31.23)	34.17 (± 24.33)	0.5655	39.85 (± 27.70)	42.83 (± 32.54)	0.7615
6 months	44.00 (± 30.33)	39.17 (± 21.16)	0.5655	56.10 (± 34.12)	48.39 (± 29.13)	0.4331
Domain	Limitation by emotional aspects			Mental health		
Before	6.65 (± 23.18)	18.39 (± 28.40)	0.3118	43.70 (± 25.65)	39.11 (± 21.24)	0.5348
1 week	26.50 (± 36.68)	31.39 (± 41.93)	0.6731	46.00 (± 21.69)	48.00 (± 22.29)	0.6059
1 month	19.95 (± 38.03)	20.33 (± 38.15)	0.9736	46.00 (± 19.68)	46.89 (± 24.54)	0.7141
3 months	28.40 (± 44.92)	9.17 (± 18.96)	0.1002	41.37(± 26.09)	42.22 (± 17.95)	0.9325
6 months	28.25 (± 42.20)	18.44 (± 32.75)	0.3979	45.10 (± 25.34)	48.00 (± 18.06)	0.6755

Orthogonal contrasts in the mixed-effects regression model

*IA* injection of local anesthetic, *TUS* therapeutic ultrasound, *SD* standard deviation*,* P*, *p* value

When comparing the VAS and NCS scores between treatment sessions, it was possible to observe a significant improvement in all treatment sessions, for both injection of local anesthetic and TUS. We observed mainly in the TUS group an important reduction in pain in the first 4 treatment sessions.

Treatment with TUS was shown to be equally effective with local anesthetic injection treatment in reducing clinical pain and improving quality of life at all times of reassessment, with no statistically significant difference between groups (*P* > 0.05).

In Tables 5 and 6, we compare the VAS and NCS scores between the treatment sessions of both groups.

**Table 5:** Evolution of clinical pain during local anesthetic injection treatment sessions measured using the visual analog and categorical scales

IA	VAS–mean (SD)	P*	NCS–mean (SD)	P*
1ª session	6.70 (± 1.53)	–	2.30 (± 0.86)	–
2ª session	4.75 (± 2.79)	0.1125	1.35 (± 0.93)	0.0011
3ª session	3.70 (± 3.15)	0.3635	1.05 (± 0.94)	0.2963
4ª session	4.30 (± 3.03)	< .0001	1.60 (± 1.39)	0.0563

Orthogonal contrasts in the mixed-effects regression model

*IA* injection of local anesthetic, *TUS* therapeutic ultrasound, *SD* standard deviation, P* *p* value

**Table 6:** Evolution of clinical pain during treatment sessions of therapeutic ultrasound injection measured using the Visual Analog and Numerical Categorical Scales

TUS	VAS–mean (SD)	P*	NCS–mean (SD)	P*
1ª session	7.22 (± 3.39)	–	3.39 (± 1.09)	–
2ª session	4.17 (± 2.66)	< .0001	1.50 (± 0.92)	< .0001
3ª session	4.33 (± 3.73)	0.8106	1.83 (1.86)	0.2710
4ª session	2.72 (± 3.29)	0.0213	1.00 (± 1.19)	0.0063
5ª session	3.11 (± 3.53)	0.5761	1.11 (± 1.23)	0.7133
6ª session	3.11 (± 3.45)	1.0000	1.06 (± 1.16)	0.8542
7ª session	2.22 (± 3.17)	0.2020	0.94 (± 1.35)	0.7133
8ª session	3.28 (± 3.48)	0.1300	1.22 (± 1.31)	0.3588
9ª session	2.78 (± 3.44)	0.4723	1.06 (± 1.35)	0.5817
10ª session	2.56 (± 3.17)	0.7493	0.89 (± 1.13)	0.5817

Orthogonal contrasts in the mixed-effects regression model

*IA* injection of local anesthetic, *TUS* therapeutic ultrasound, *SD* standard deviation, P* *p* value

In a post-hoc power analysis, performed in the G*Power program version 3.1.9.2, assuming an effect size of 0.25 with a significance level of 5%, with 2 groups and 5 repeated measurements of the same patient, and a sample size of 19, the power of the test was 76%.

## Discussion

### Main findings

TUS is as effective as the injection of local anesthetic in the treatment of women with AMPS associated with CPP.

### Interpretation of results

The studies of Ilter [33]⁠, Sarrafzadeh [45]⁠, Rai [41]⁠, and Kim [46]⁠ have already shown that TUS can be effective in the treatment of myofascial MTrP. These previously described data corroborated the data from our study, but none of them demonstrated the efficacy of TUS in MTrP from AMPS. With the results found in our research, TUS can be considered a form of treatment for CPP secondary to AMPS.

The effectiveness found in our study can be explained, at least partially, by the thermal and nonthermal (mechanical) effects of the TUS. The thermal effects are increased blood flow. The nonthermal effects include increased vascular permeability, blood flow and fibroblastic activity, tissue regeneration, increased speed of motor and sensory nerve conduction, and reduced muscle spasms. These effects could justify the improvement of AMPS symptoms [47–49]⁠⁠.

When a physical or pharmacological treatment is performed, many things can explain the clinical improvement of patients; the placebo effect is one of them, and scientific evidence has shown that it exists. Medicines or physical agents applied are important, but we must also integrate other elements, such as the therapist-patient relationship. This is a true biopsychosocial phenomenon produced by the context in which an intervention is carried out. Placebo and nocebo responses are changes in patients’ symptoms due to their participation in the therapeutic meeting. This infinity of signs inherent in any intervention is perceived and interpreted by patients and can create positive or negative expectations [50]⁠. There are also other effects that could influence the clinical improvement of patients, one of which is the Hawthorne effect, which is described as the act of participating in a clinical trial that can produce an improvement in symptoms due to the observation that the patient receives from researchers [47]⁠.

In some studies, adverse events were found during treatment with TUS in experimental models, but no serious adverse events in humans have been reported. These adverse events in experimental models were cell lysis, increased free radical formation, and decreased blood flow due to constriction of arterioles forming thrombi [51–55]⁠. The study by Mengi et al. [55]⁠ evaluated whether the TUS caused changes in renal function in humans undergoing treatment in the lumbar region, and it was found that the TUS did not change the renal function of patients. This study brings us security because it was a treatment performed very close to the renal region, and even then there was no change in the organ function, but the authors did not rule out that elderly patients with impaired renal function should be closely monitored when undergoing this type of treatment, and further studies in larger samples with different ages were also suggested to reach more robust conclusions.

The injection of local anesthetic is a treatment with proven efficacy in AMPS [22, 25] and it is believed that the effect of the local anesthetic occurs through the interruption of excitation and nerve conduction by direct interaction with the sodium channels, thus generating a reduction in inflammation and activation of acetylcholine at the neuromuscular junction. However, it is a treatment where some undesirable side effects can be found such as skin infection, injection needle breakage, hematoma formation, vasovagal syncope, and myotoxicity [12, 14, 56, 57] ⁠. In our research, no adverse events were reported in both treatment groups.

In the study by Aguilera et al. [58]⁠, it was found that ischemic compression and TUS are effective in the treatment of latent MTrP of the trapezius muscle, but ischemic compression was superior to TUS in long-term results. In the study by Montenegro et al. [22], it was found that injection of local anesthetic is superior to local ischemic compression in patients with AMPS. MTrPs are also found in different places, also showing differences in their treatment.

### Limitations

The first limitation of the study was the absence of blinding; however, due to the differences in the forms of treatment, this limitation cannot be minimized, but we emphasize that the data analysis was performed by a researcher blinded to the types of treatment. The second limitation is the exclusion of women with comorbidities and other causes of CPP. Considering this fact, we cannot affirm that both interventions work in the same way, since most women followed up in a clinic specializing in chronic pain have associated comorbidities and more than one painful region that can also be justified by the somatization process, but these exclusions were necessary to avoid bias in the interpretation of the results. The absence of a placebo group was the third limitation, but the ethical implications of not treating a patient with chronic pain prevented us from forming this group. The fourth limitation presented in this study can be considered the difference between the number of sessions used for each technique, influenced by the long interaction and doctor-patient relationship in TUS sessions. The COVID-19 pandemic was the fifth limitation presented in the study, because with the pandemic, outpatient care was suspended, thus preventing data collection and recruitment of new patients.

## Conclusion

TUS was as effective as the injection of local anesthetic in reducing pain and improving the quality of life of patients with CPP secondary to AMPS; however, further studies should be carried out with a larger number of patients and with a longer follow-up.

## Data Availability

All data generated or analysed during this study are included in this published article.

## Abbreviations

CPP: Chronic pelvic pain
AMPS: Abdominal myofacial pain syndrome
MTrPS: Hyperirritable trigger points
TENS: Transcutaneous electrical nerve stimulation
TUS: Therapeutic ultrasound
IA: Injection of local anesthetic
VAS: Visual analogic scale
NCS: Numerical categorical scale
